# Eupatilin Inhibits Renal Cancer Growth by Downregulating MicroRNA-21 through the Activation of YAP1

**DOI:** 10.1155/2019/5016483

**Published:** 2019-04-24

**Authors:** Weifeng Zhong, Zhiming Wu, Nanhui Chen, Kaihua Zhong, Yifeng Lin, Huiming Jiang, Pei Wan, Shanming Lu, Lawei Yang, Siping Liu

**Affiliations:** ^1^Department of Urology, Meizhou People's Hospital, Meizhou 514031, China; ^2^Department of Urology, Sun Yat-sen University Cancer Center, State Key Laboratory of Oncology in South China, Collaborative Innovation Center for Cancer Medicine, Guangzhou 510060, China; ^3^Department of Pathology, School of Basic Medical Sciences, Southern Medical University, Guangzhou 510515, China

## Abstract

Renal cell carcinoma (RCC) is the second most common human urinary tumor. Eupatilin is the main active ingredient of the traditional Chinese medicine* Artemisia asiatica*. The effect of Eupatilin on RCC and the underlying mechanism remain unknown. Here, we investigated the anticancer effects and mechanisms of Eupatilin in RCC* in vivo* and* in vitro*, laying an experimental foundation for the clinical application of Eupatilin in the treatment of RCC. The results showed that Eupatilin significantly inhibited 786-O cell viability and migration and promoted apoptosis. Eupatilin inhibited the expression of miR-21 in 786-O cells, and overexpression of miR-21 suppressed the effect of Eupatilin on viability, apoptosis, and migration in 786-O cells. Eupatilin inhibited the growth of renal tumors in nude mice by downregulating miR-21. YAP1, which was identified as a target of miR-21, showed significantly lower expression in RCC tissues than in healthy tissues. miR-21 significantly inhibited YAP1 protein expression in 786-O cells and tumor tissues isolated from nude mice, and YAP1 attenuated the effect of miR-21 on the viability, apoptosis, and migration of 786-O cells. In conclusion, Eupatilin inhibited the expression of miR-21, which mediated the proapoptotic and antimigratory effects of Eupatilin by suppressing YAP1 in renal cancer cells. These results suggested that Eupatilin could be a potent agent for the treatment of RCC.

## 1. Introduction

Renal cell carcinoma (RCC), also known as renal adenocarcinoma or renal cancer, is a malignant tumor that originates in the urinary tubule epithelial system of the renal parenchyma. RCC includes various renal cell carcinoma subtypes originating in different parts of the urinary tubule, whereas it does not include tumors derived from the renal interstitial and epithelial systems [1]. Renal cancer accounts for approximately 90% of all primary renal malignancies and is a common urological tumor. RCC is the second most common human urinary tumor and one of the most common malignancies worldwide. The incidence rate of RCC increases yearly, and RCC has become a major disease that seriously threatens human health [2]. Renal cancer itself is a multi-gene-related tumor, the pathogenesis of which is extremely complex, and the molecular biological basis of disease progression remains to be elucidated [3]. In addition to tissue resection, drug therapy plays an important role in controlling RCC development and preventing metastasis. Under the background of internationalization of new drug development for the treatment of renal cancer, many effective chemically synthesized drugs have been identified; however, the toxic effects of these agents on normal cells cannot be ignored, and their use is limited by the patient's physical condition [4]. The use of natural medicine ingredients to treat diseases not only reduces production costs but also reduces toxicity to the human body. In addition, the development of new medicinal ingredients contributes to overcoming the multidrug resistance (MDR) phenomenon associated with the clinical treatment of tumors [5]. Improving our understanding of the mechanisms underlying the effects of natural medicines such as traditional Chinese medicine could be useful for the synthesis of drugs with low toxicity [6]. Compared with the exploration of new substances, the use of natural medicines is associated with greater cost-effectiveness and improved resource allocation.

Eupatilin is an O-methyl-flavonoid with the molecular formula C_18_H_16_O_7_. It is an important flavonoid and an active ingredient of the leaves of Ai Ye, a traditional Chinese medicine [7]. The role of this natural substance in the treatment of human diseases has been investigated extensively [8–10]. Studies show that Eupatilin has a therapeutic effect on endometrial cancer and can inhibit the proliferation of human endometrial cancer cells by upregulating the expression of P21 and inducing G2/M cell cycle arrest [11]. However, there is little research on Eupatilin in renal cancer. Previous work from our group showed that Eupatilin inhibits the proliferation of renal cancer cells, although the underlying mechanism in RCC remains unclear [12].

In the present study, we showed that Eupatilin inhibited cell proliferation and migration and promoted apoptosis in association with the downregulation of miR-21 in 786-O cells and in renal tissues of patients with RCC. A molecular biology approach was used to investigate the relationship between RCC cell apoptosis induced by Eupatilin and the miR-21–yes-associated protein 1 (YAP1) signaling axis. The miR-21-YAP1 signaling axis regulated by Eupatilin may become a new therapeutic target in RCC, providing new ideas for traditional Chinese medicine and targeted therapy in RCC.

## 2. Materials and Methods

### 2.1. Cell Culture and Treatment

Human renal cancer 786-O cells were obtained from Shanghai Cell Institute Country Cell Bank (Shanghai, China). 786-O cells were grown in DMEM supplemented with 10% fetal bovine serum (FBS) (Gibson/BRL, MD, USA), 100 U/mL penicillin G, and 100 *μ*g/mL streptomycin (Sigma-Aldrich Corp., St. Louis, MO, USA). Cells were maintained at 37°C in a humidified 5% CO_2_ incubator. For Eupatilin (Sigma, USA) treatment, cells were treated with different concentrations of Eupatilin (5, 10, and 20 mM) for 24 h.

### 2.2. Cell Transfection

The cell transfection was performed as described by* Yang et al.* [13]. miR-NC and miR-21 mimics were obtained from Gene Pharma Co. Ltd (China). For transfection, 786-O cells were transfected with 50 pmol/mL miR-21 mimics or miR-NC using Lipofectamine 2000 (Invitrogen) according to the manufacturer's instructions. For YAP1 functional assays, a YAP1 expression vector was generated by cloning the open reading frame of the YAP1 gene into the pcDNA3.1 vector, and pcDNA3.1-YAP1 plasmids lacking the 3′ untranslated region (3′-UTR) were cotransfected with miR-21 mimics into cells.

### 2.3. Tumorigenicity Assays in Nude Mice

Six-week-old male BALB/c athymic nude mice were subcutaneously injected in the right armpit region with 1.5 × 10^6^ cells in 0.1 mL PBS. Three groups of mice (n = 6/group) were tested. Group 1 (Control) and group 2 (Eupatilin) were injected with 786-O cells infected with negative control miRNA; group 3 (Eupatilin+miR-21) was injected with 786-O cells infected with miR-21 mimics. When tumors were established (at 1–2 weeks), mice in groups 2 and 3 were injected with Eupatilin (10 mg/kg) every 7 days. Mice in group 1 received intramuscular injections of PBS as a control. Tumor size was measured every 7 days with calipers, and tumor volume was calculated using the following formula: (L × W2)/2, where L is the length and W is the width of the tumor. Mice were killed at 4 weeks, and tumor weight was measured. All experimental procedures involving animals were performed in accordance with the Guide for the Care and Use of Laboratory Animals (NIH publication no. 80-23, revised 1996) and according to the Institutional Ethical Committee of Animal Care in Sun Yat-sen University.

### 2.4. Cell Viability Assay

Cell processing and viability assays are as described in our previous article [12]. The cells were seeded into 96-well plates at a density of 5 × 10^3^ cells per well and treated with various concentrations of Eupatilin (5, 10, and 20 mM). After the cells were treated with Eupatilin for 24h, 10 *μ*L of CCK-8 reagent was added and incubated for 2 h at 37°C. The optical density (OD 450) value was read. The viability rate of cells was calculated as the OD values of treated groups/the OD values of the control group × 100%.

### 2.5. Apoptosis Assay

The cell apoptosis assay was performed as described by* Yang et al.* [13]. The cells treated with Eupatilin were washed and resuspended in detection buffer. The cells were then incubated with 10 *μ*L of Annexin V-FITC and 5 *μ*L of PI dye solution for 8 min at room temperature in the dark. The apoptosis rate was measured by flow cytometry.

### 2.6. Western Blotting

Cells were lysed on ice in lysis buffer (Beyotime, China) supplemented with protease and phosphatase inhibitors (Roche, USA) and 1 mM PMSF (Sigma, USA) and centrifuged at 12000 rpm for 30 min at 4°C. Proteins were separated by 10–12% SDS-PAGE and transferred to PVDF membranes. After blocking in 5% nonfat milk, the membranes were incubated with primary antibodies against YAP1 (Abcam, USA) and tubulin (Abcam) diluted at 1:1000, followed by washing in TBST and incubation with goat anti-rabbit HRP-conjugated secondary antibodies (1:5000) at room temperature for 1 h. Protein bands were visualized with enhanced chemiluminescence reagents (Pierce, USA).

### 2.7. Transwell Assays

The cell migration assay was performed as described by* Wang et al.* [14]. Briefly, 2 × 10^4^ 786-O cells were seeded into the upper chamber of the Transwell filter. DMEM complete medium (10% FBS) containing Eupatilin was added to the lower culture plate. After incubation for 24 h, the invaded cells were fixed and stained with crystal violet dye solution. Photographs of five randomly selected fields of the fixed cells were taken and cells were counted under an inverted light microscope (Leica, Germany).

### 2.8. Quantitative Real-Time PCR (QRT-PCR)

The qRT-PCR assay was performed as described by* Hang et al.* [15]. Total RNA was extracted from clinical samples and 786-O cells treated with Eupatilin using the TRIzol reagent (Ambion®) according to the manufacturer's protocol. RNA samples (1 *μ*l per sample) were reverse-transcribed into cDNA according to the manufacturer's protocol. U6 was used as an endogenous reference. The expression of miR-21 and U6 was detected by 2-step PCR. Primers for miR-21 and U6 were as follows: miR-21-F, 5′-GCGGCGGTAGCTTATCAGACTG-3′; miR-21-R, 5′-ATCCAGTGCAGGGTCCGAGG-3′; U6-F, 5′-CGCTTCGGCAGCCACATATACTA-3′, U6-R, 5′-CGCTTCACGAATTTGCGTGTCA-3′. All experiments were performed in duplicate and repeated twice. Results are presented as fold induction calculated using the 2^-∆∆Ct^ method.

### 2.9. Luciferase Assays

The putative binding sites of miR-21 in the 3′-UTR of the human YAP1 gene were amplified and inserted into the luciferase reporter psiCHECK vector. Mutations were introduced into the 3′-UTR of YAP1 (UUCGAAUA to UUAUGAUA) using a QuikChange Site-Directed Mutagenesis Kit (Stratagene). 786-O cells were seeded in 96-well plates at 6000 cells per well the day before transfection. A mixture of 100 ng psi-YAP1 3′-UTR and 200 ng of NC or miR-21 mimics was transfected into 786-O cells using Lipofectamine 2000. After 48 h, Firefly and Renilla luciferase activities were measured with a Dual-Luciferase Reporter System (Promega) according to the manufacturer's protocol.

### 2.10. Statistical Analysis

Data are expressed as the mean ± SEM unless otherwise noted. The two-tailed Student's* t*-test was used to analyze the differences between two groups for cells and nonparametric tests were used to analyze the differences between two groups for tissues. A P value <0.05 was considered statistically significant.

## 3. Results

### 3.1. Effect of Eupatilin on RCC Cell Proliferation, Apoptosis, and Migration* In Vitro*

**Figure 1:**
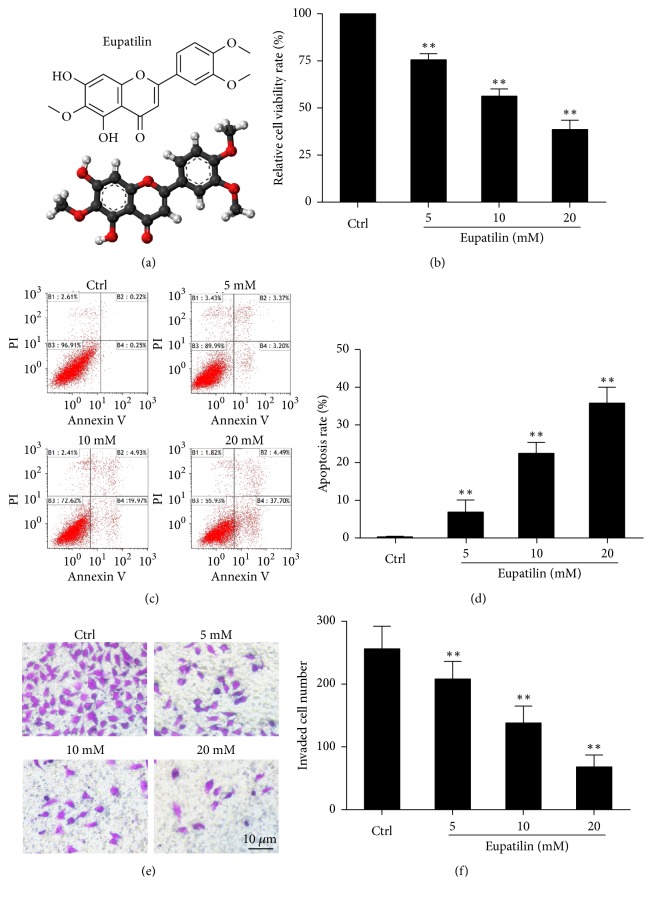
*Effect of Eupatilin on proliferation, apoptosis, and migration of renal cell carcinoma in vitro*. (a) The molecular structure of Eupatilin. (b) 786-O cells were treated with Eupatilin (5, 10, or 20 mM) for 24 h. Cell viability was detected with the CCK8 assay. Data are expressed as the mean ± SEM (n = 3) of three independent experiments. (*∗∗P* < 0.01 versus control). (c) 786-O cells were treated with Eupatilin (5, 10, or 20 mM) for 24 h. The apoptosis of 786-O cells was detected by flow cytometry. (d) Quantification of the results of (c). Data are expressed as the mean ± SEM (n = 3) of three independent experiments. (*∗P* < 0.05 and *∗∗ P* < 0.01 versus control). (e) 786-O cells were treated with Eupatilin (5, 10, or 20 mM) for 24 h. The migration ability of 786-O cells was detected with the Transwell assay. (f) Quantification of the results in (e). Data are expressed as the mean ± SEM (n = 3) of three independent experiments. (*∗∗P* < 0.001 versus control).

Previous work from our group showed that Eupatilin inhibits the proliferation of renal cancer cells [12]. To confirm this finding, we examined the effect of Eupatilin on proliferation, apoptosis, and migration of renal cancer cells. The molecular structure of Eupatilin is shown in Figure 1(a). Eupatilin significantly inhibited the viability of 786-O cells (*P *< 0.05) (Figure 1(b)) and significantly promoted 786-O cell apoptosis (*P* < 0.05) (Figures 1(c) and 1(d)) in a concentration-dependent manner, as shown by the CCK-8 assay and flow cytometry, respectively. As shown in Figures 1(e) and 1(f), the number of cells invading across the polycarbonate membrane was significantly higher in the Eupatilin-treated group than in the control group (*P* < 0.05). These results confirm that Eupatilin inhibits RCC progression* in vitro*.

### 3.2. Eupatilin Inhibits miR-21 Expression in Renal Cancer Cells

**Figure 2:**
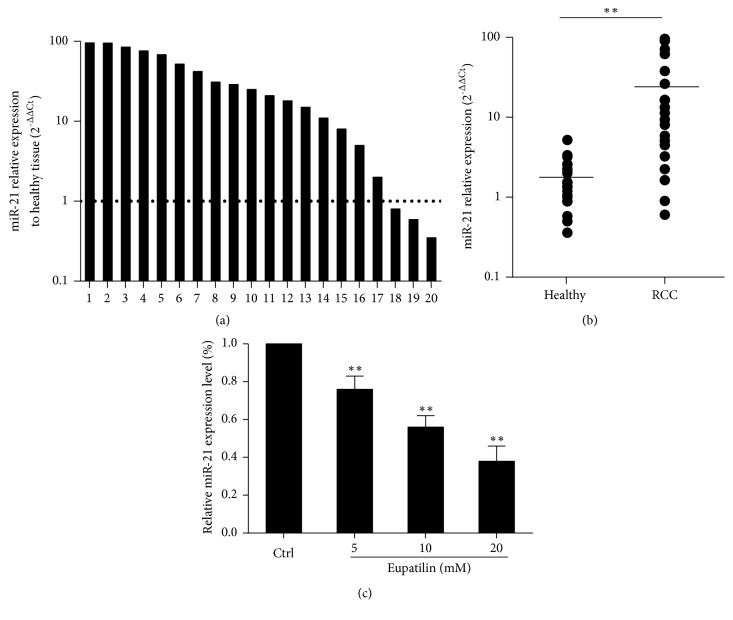
*Eupatilin downregulates miR-21 in renal cancer cells*. (a) miR-21 expression assessed by qRT-PCR in 20 renal cancer tumor specimens (RCC) relative to that in normal adjacent renal tissues (healthy). (b) Quantification of the results in (a). Data are expressed as the mean ± SEM (n = 3) of three independent experiments. (*∗∗P* < 0.01 versus healthy). (c) 786-O cells were treated with Eupatilin (5, 10, or 20 mM) for 24 h. The relative expression of miR-21 in 786-O cells was assessed by qRT-PCR. Data represent the mean ± SEM (n = 3) of three independent experiments. (*∗∗P* < 0.01 versus control).

Furthermore, miR-21 expression was measured in 20 RCC tissues and 20 normal tissues by quantitative RT-PCR (qRT-PCR), which showed that miR-21 expression was higher in RCC tissues than in normal tissues (Figures 2(a) and 2(b)). The effect of Eupatilin on miR-21 in RCC was examined by qRT-PCR detection of miR-21 expression in Eupatilin-treated 786-O cells, which showed that Eupatilin significantly downregulated miR-21 in a concentration-dependent manner (*P* < 0.05) (Figure 2(c)). These results indicate that the inhibitory effect of Eupatilin on the growth of RCC may be related to the downregulation of miR-21 expression.

### 3.3. miR-21 Overexpression Suppresses Eupatilin-Induced Apoptosis and Migration Inhibition in RCC Cells* In Vitro*

**Figure 3:**
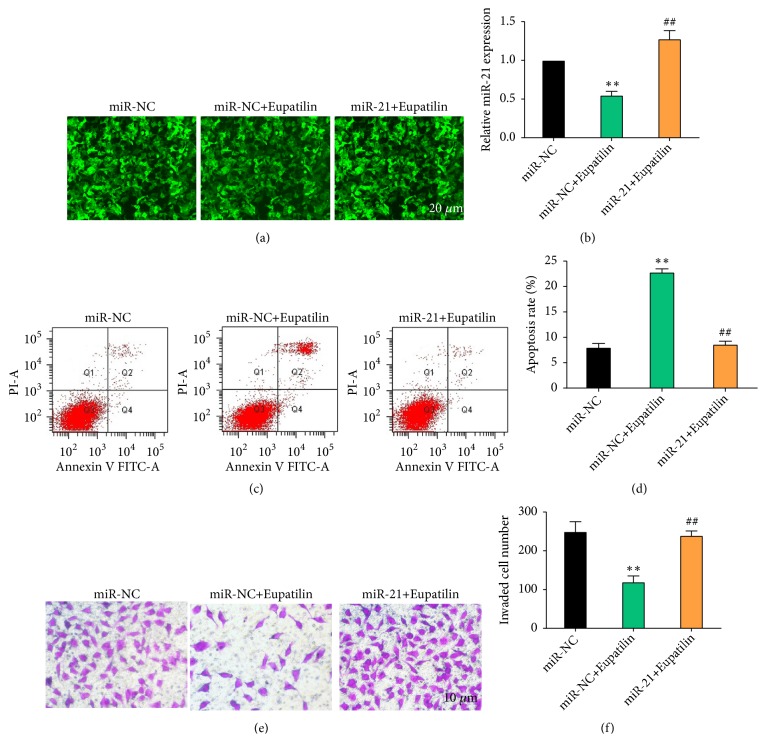
*miR-21 overexpression suppresses Eupatilin-induced apoptosis and migration inhibition in renal cancer cells in vitro*. (a) 786-O cells infected with miR-21 mimics or control lentivirus were treated with Eupatilin (10 mM) for 24 h. Fluorescent image of renal cancer 786-O cells showing the efficacy of lentivirus infection (Green: GFP). (b) 786-O cells infected with miR-21 mimics or control lentivirus were treated with Eupatilin (10 mM) for 24 h. The relative expression of miR-21 in 786-O cells was assessed by qRT-PCR. Data represent the mean ± SEM (n = 3) of three independent experiments. (*∗∗P* < 0.01 versus miR-NC; ^##^* P* < 0.01 versus miR-NC+Eupatilin). (c) 786-O cells infected with miR-21 mimics or control lentivirus were treated with Eupatilin (10 mM) for 24 h. The apoptosis of 786-O cells was detected by flow cytometry. (d) Quantification of the results in (c). Data represent the mean ± SEM (n = 3) of three independent experiments. (*∗∗P* < 0.01 versus miR-NC; ^##^* P* < 0.01 versus miR-NC+Eupatilin). (e) 786-O cells infected with miR-21 mimics or control lentivirus were treated with Eupatilin (10 mM) for 24 h. The migration ability of 786-O cells was detected with the Transwell assay. (f) Quantification of the results in (e). Data represent the mean ± SEM (n = 3) of three independent experiments. (*∗∗P* < 0.01 versus miR-NC; ^##^* P* < 0.01 versus miR-NC+Eupatilin).

To determine whether miR-21 downregulation was involved in Eupatilin-induced apoptosis in renal cancer cells, miR-21 was overexpressed by mimic transfection, and apoptosis and migration were measured. qRT-PCR analysis showed that miR-21 was significantly upregulated in the miR-21 mimics group compared with the control (miR-NC) group (Figures 3(a) and 3(b),* P* < 0.01). miR-21 overexpression significantly decreased Eupatilin-induced apoptosis in 786-O cells (Figures 3(c) and 3(d),* P* < 0.01) and restored the Eupatilin-induced inhibition of 786-O cell migration (Figures 3(e) and 3(f),* P* <0.01). These results indicate that miR-21 overexpression blocked the effect of Eupatilin on apoptosis and migration in renal cancer cells.

### 3.4. miR-21 Overexpression Suppresses Eupatilin-Induced Growth Inhibition in RCC* In Vivo*

**Figure 4:**
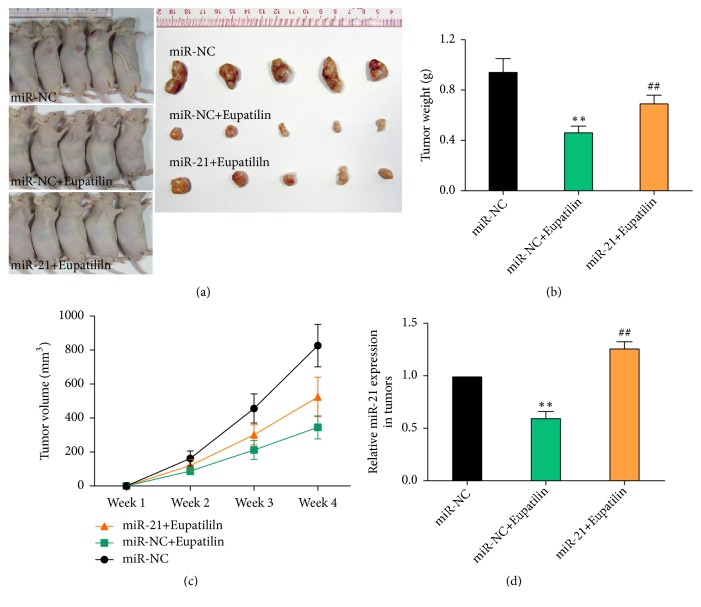
*miR-21 overexpression suppresses Eupatilin-induced growth inhibition of renal cancer cells in vivo*. (a) Images of nude mice injected with 786-O cells infected with miR-21 mimics or control lentivirus and tumors isolated from the mice after 35 days. Images are representative of the results obtained from six mice. (b) Mice injected with 786-O cells infected with miR-21 mimics or control lentivirus were treated with Eupatilin. After the mice were killed, tumor tissues were excised and tumor weight was measured. Data represent the mean ± SEM of six mice per group. (*∗∗P* < 0.01 versus miR-NC; ^##^* P* < 0.01 versus miR-NC+Eupatilin). (c) Growth curves of tumors resulting from injection of 786-O cells into nude mice. Tumor volumes were estimated using calipers. Data represent the mean ± SEM of six mice per group. (*∗∗P* < 0.01 versus miR-NC; ^##^* P* < 0.01 versus miR-NC+Eupatilin). (d) Nude mice injected with 786-O cells infected with miR-21 mimics or control lentivirus were treated with Eupatilin. The relative expression of miR-21 in tumors was assessed by qRT-PCR. Data represent the mean ± SEM of six mice per group. (*∗∗P* < 0.01 versus miR-NC; ^##^* P* < 0.01 versus miR-NC+Eupatilin).

The effect of Eupatilin on cell growth* in vitro* was examined in an* in vivo* model system established by injecting miR-21-overexpressing or negative control 786-O cells into nude mice subcutaneously. In mice treated with Eupatilin, tumor proliferation was inhibited and tumor volume was significantly smaller than that in the control group. These results indicate that Eupatilin can inhibit the growth of renal cancer cells* in vivo*. In mice with miR-21 overexpressing tumors, Eupatilin treatment promoted proliferation and tumor volume was significantly greater than that in the control group treated with Eupatilin (*P* < 0.01) (Figure 4). These results indicate that miR-21 suppressed Eupatilin-induced tumor growth inhibition in nude mice.

### 3.5. YAP1 Is a Direct Target of miR-21 in Renal Cancer Cells

**Figure 5:**
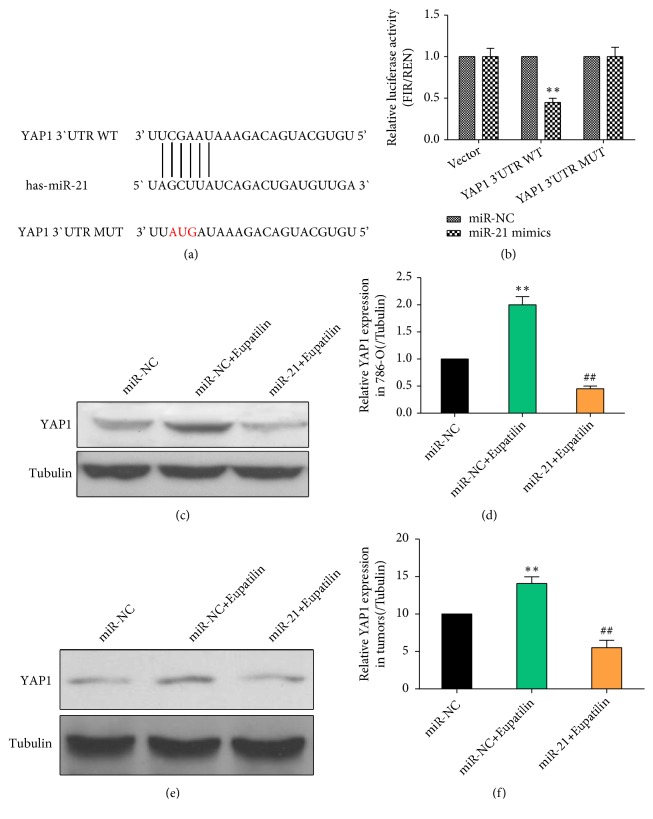
*YAP1 is a direct target of miR-21 in renal cancer cells.* (a) Schematic representation of YAP1 mRNA with a putative 3′-UTR miR-21-binding site and sequences of wild-type (YAP1 WT) and mutant (YAP1 MUT) miR-21 target sites. The putative miR-21 binding site in the YAP1 3′-UTR is indicated in red. This site was identified using TargetScan. (b) Luciferase reporter assay of 786-O cells transfected with a vector with an inserted YAP1 3′-UTR sequence (YAP1 3′-UTR WT) or a vector with an inserted mutated 3′-UTR sequence (YAP1 3′-UTR MUT), together with negative control miRNA (miR-NC) or miR-21 mimics. Luciferase activity was normalized to Renilla luciferase activity. Data represent the mean ± SEM (n = 3) of three independent experiments. (*∗∗P* < 0.01 versus miR-NC). (c) 786-O cells infected with miR-21 mimics or control lentivirus were treated with Eupatilin (10 mM) for 24 h. The protein expression of YAP1 in 786-O cells was detected by western blotting. (d) Quantification of the results in (c). Data represent the mean ± SEM (n = 3) of three independent experiments. (*∗∗P* < 0.01 versus miR-NC; ^##^* P* < 0.01 versus miR-NC+Eupatilin). (e) Mice injected with 786-O cells infected with miR-21 mimics or control lentivirus were treated with Eupatilin. The protein expression of YAP1 in tumors was assessed by western blotting. (f) Quantification of the results in (e). Data represent the mean ± SEM (n = 3) of three independent experiments. (*∗∗P* < 0.01 versus miR-NC; ^##^* P* < 0.01 versus miR-NC+Eupatilin).

To elucidate the mechanisms by which miR-21 inhibited tumor growth, the online program TargetScan was used to predict miR-21 targets. The putative miR-21 binding site was identified within the 3′-UTR of YAP1 mRNA by TargetScan analysis (Figure 5(a)). To test the direct binding of miR-21 to this site, wild-type or mutant full-length YAP1 3′-UTR luciferase reporter constructs were generated and cotransfected with miR-21 mimics into 786-O cells. Luciferase activity was significantly inhibited by miR-21 mimics in 786-O cells transfected with wild-type YAP1 constructs compared with that in controls, indicating that miR-21 can directly interact with the 3′-UTR of YAP1 mRNA (Figure 5(b)). Assessment of the effect of miR-21 on endogenous YAP1 protein expression in 786-O cells showed that miR-21 overexpression significantly downregulated YAP1 (Figures 5(c) and 5(d)). Similar results were observed in tumor tissues isolated from nude mice injected subcutaneously with 786-O cells (Figures 5(e) and 5(f)). These results indicate that miR-21 negatively regulated YAP1 by targeting a specific site in the YAP1 3′-UTR.

### 3.6. Potential Role of the YAP1-AKT Signaling Pathway in Eupatilin-Induced miR-21 Downregulation* In Vitro*

**Figure 6:**
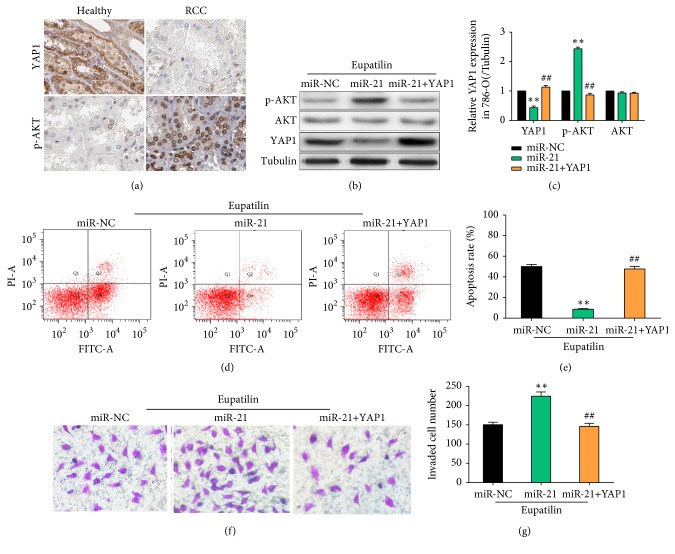
*Potential role of the YAP1-signaling pathway in miR-21 reduction by Eupatilin in vitro. *(a) The expression of YAP1 and p-AKT was detected by immunohistochemistry in 20 pairs of renal cell carcinoma and healthy tissues. Representative images are shown. (b) 786-O cells were infected with negative control lentivirus (miR-NC), miR-21 mimics (miR-21), or miR-21 plus YAP1 (miR-21+YAP1) and treated with Eupatilin for 24 h. The expression of YAP1, p-AKT, and AKT in 786-O cells was assessed by western blotting. (c) Quantification of the results in (b). Data represent the mean ± SEM (n = 3) of three independent experiments. (*∗∗P* < 0.01 versus miR-NC; ^##^* P* < 0.01 versus miR-21). (d) 786-O cells were infected with negative control lentivirus (miR-NC), miR-21 mimics (miR-21), or miR-21 plus YAP1 (miR-21+YAP1) and treated with Eupatilin for 24 h. The apoptosis of 786-O cells was detected by flow cytometry. (e) Quantification of the results in (d). Data represent the mean ± SEM (n = 3) of three independent experiments. (*∗∗P* < 0.01 versus miR-NC; ^##^* P* < 0.01 versus miR-21). (f) 786-O cells were infected with negative control lentivirus (miR-NC), miR-21 mimics (miR-21), or miR-21 plus YAP1 (miR-21+YAP1) and treated with Eupatilin for 24 h. The migration ability of 786-O cells was detected using the Transwell assay. (g) Quantification of the results in (f). Data represent the mean ± SEM (n = 3) of three independent experiments. (*∗∗P* < 0.01 versus miR-NC; ^##^* P* < 0.01 versus miR-21).

To confirm the role of YAP1 in RCC, YAP1 protein expression was assessed by immunohistochemistry, which showed that YAP1 protein levels were significantly lower in RCC tissues than in healthy tissues (Figure 6(a)). In addition, the expression of p-AKT was significantly higher in RCC tissues than in healthy tissues. To confirm that YAP1 was involved in the effect of miR-21 on promoting renal cancer cell growth, we investigated whether exogenous expression of YAP1 affected the phosphorylation of AKT and the effects of miR-21 in 786-O cells. Cells were infected with negative control lentivirus (control), miR-21 mimics (miR-21), or miR-21 plus YAP1 (miR-21+YAP1), and apoptosis and migration were assessed after Eupatilin treatment. Western blot analysis showed that YAP1 expression was significantly lower, whereas p-AKT was significantly higher in the miR-21 group than in the miR-NC group. YAP1 protein expression was significantly higher, whereas p-AKT was significantly lower in the miR-21 + YAP1 group than in the miR-21 group (Figures 6(b) and 6(c)). Cells infected with miR-21 mimics alone showed reduced apoptosis rates, as determined by flow cytometry; however, coinfection with YAP1 lentivirus reversed this effect (Figures 6(d) and 6(e)). Similar results were observed in Transwell assays, in which cells infected with miR-21 mimics showed increased migration ability, and coinfection with YAP1 lentivirus reversed this effect (Figures 6(f) and 6(g)). These results confirm the hypothesis that exogenous expression of YAP1 affects the phosphorylation of AKT and compromises the effects of miR-21 in 786-O cells.

## 4. Discussion

Renal cancer accounts for approximately 90% of all primary malignant renal tumors and ranks second among human urinary system tumors. Epidemiological studies show that the incidence of renal cancer increases yearly, and RCC has become a major threat to human health [16]. Therefore, identifying natural medicines with therapeutic efficacy is an important research topic.

In the present study, Eupatilin suppressed the proliferation of renal cancer cells* in vitro* and* in vivo*. Eupatilin downregulated miR-21 and suppressed the expression of YAP1, ultimately leading to cell apoptosis of renal cancer cells. Increased expression of miR-21 inhibits apoptosis in renal cancer cells, encountering the proapoptotic effect of Eupatilin. Overall, the present findings demonstrated that Eupatilin has a proapoptotic effect on renal cancer cells, and the contribution of miR-21 to this biological process was elucidated. These results suggested that Eupatilin could be a new therapeutic agent for the treatment of renal cancer.

Eupatilin is an O-methyl-flavonoid and its molecular formula is C_18_H_16_O_7_. It is an important flavonoid active ingredient of the* Artemisia asiatica* (Compositae). It is commonly used to treat acid-base imbalances in the body [17]. Eupatilin has an inhibitory effect on a variety of tumors. Eupatilin induces apoptosis of osteosarcoma U-2OS cells through mitochondrial endogenous pathways and inhibits the proliferation of osteosarcoma cells [18]. Eupatilin has a significant therapeutic effect on gastric cancer, and it inhibits the growth of gastric cancer cells by blocking STAT3-mediated vascular endothelial growth factor expression [19]. Eupatilin decreases nuclear factor *κ*B activity and inhibits the invasion and proliferation of human gastric cancer MKN-1 cells [20]. However, there is little research on Eupatilin in renal cancer, and the mechanism of action in RCC remains unclear. The present results confirmed the anticancer role of Eupatilin* in vitro* and* in vivo* by showing that Eupatilin suppressed renal cancer cell growth by modulating miR-21 and YAP1 expression.

Clinical studies show that changes in tumor cell migration and invasion directly affect the process of tumor metastasis [21–23]. Therefore, identifying key drugs and molecular targets to inhibit cell migration and invasion is important for the prevention and treatment of tumors in clinical practice. The present study confirmed the inhibitory effect of Eupatilin on the migration ability of renal cancer 786-O cells. Eupatilin inhibited the migration of 786-O cells by downregulating the expression of miR-21, suggesting the possible use of Eupatilin for the treatment or adjuvant treatment of renal cancer.

miR-21 is located on chromosome 17q23. 2 and has an autonomous transcription unit. miR-21 is one of the first studied miRNAs and regulates the expression of various tumor suppressor genes [24–26]. In the present study, qRT-PCR analysis showed that miR-21 is overexpressed in RCC, which is consistent with previous reports. To examine the role of miR-21 in the Eupatilin-mediated regulation of proliferation, apoptosis, and migration of renal cancer cells, we assessed the effect of different concentrations of Eupatilin on miR-21 expression by Q-PCR, which showed that Eupatilin significantly downregulated miR-21. Assessment of the effect of miR-21 on renal cancer cells showed that miR-21 overexpression significantly inhibited the apoptosis and antimigration effects of Eupatilin in 786-O cells. In nude mice, miR-21 overexpression significantly suppressed the inhibitory effect of Eupatilin on the tumorigenicity of renal cancer cells* in vivo*. Therefore, Eupatilin inhibited miR-21 expression, promoted apoptosis, and inhibited the migration of renal cancer cells.

The inhibitory function of miRNAs is mediated by binding to the 3′-UTR of target genes, which results in mRNA degradation [27]. Here, we identified YAP1 as a direct target of miR-21. We showed evidences that miR-21 reduced the protein expression of YAP1 and miR-21 directly binds to the 3′-UTR of YAP1. The YAP1 gene is located on chromosome 11q13. YAP has two alternative spliceosomes: YAP1 and YAP2 [28]. YAP1 expression is tissue-specific and it plays different roles in the development of different tumors [29–31]. YAP1 is a transcriptional coactivator that specifically binds to the transcription factors of oncogenes or tumor suppressor genes, thereby exerting cancer-promoting or tumor-suppressing effects [32]. YAP1 can inhibit the AKT pathway and suppress the growth of thyroid carcinoma cells [33]. In the present study, YAP1 overexpression inhibited the phosphorylation of AKT and reduced the effect of miR-21 on renal cell apoptosis and migration, supporting the hypothesis that Eupatilin inhibits the expression of miR-21 in renal cancer cells and that miR-21 regulates apoptosis and migration in RCC by targeting YAP1. The present study is the first to demonstrate that Eupatilin exerts proapoptotic and antimigratory effects on renal cancer cells via the miR-21/YAP1 signaling axis.

## 5. Conclusions

Eupatilin inhibited the expression of miR-21 via YAP1-AKT pathway in renal cancer cells. The data provide a better understanding of the molecular mechanism underlying the anticancer activity of Eupatilin in renal cancer cells and suggest that Eupatilin could be a potent agent for renal cancer treatment. Further studies are needed to validate the therapeutic potential of Eupatilin in renal cancer* in vivo*, and its effects on renal cancer should be assessed in clinical practice.

## Data Availability

The data used to support the findings of this study are available from the corresponding author upon request.

## Abbreviations

CCK8: Cell counting kit-8
FBS: Fetal bovine serum
MDR: Multidrug resistance
NC: Negative control
OD: Optical density
qRT-PCR: Quantitative real-time reverse transcription PCR
RCC: Renal cell carcinoma
UTR: Untranslated region
YAP1: Yes-associated protein 1.

## References

[1] Bandini M., Smith A., Zaffuto E. (2018). Effect of pathological high-risk features on cancer-specific mortality in non-metastatic clear cell renal cell carcinoma: a tool for optimizing patient selection for adjuvant therapy. *World Journal of Urology*.

[2] Minervini A., Campi R., Lapini A., Carini M. (2017). Morbidity of metastasectomy for renal cell carcinoma: emerging evidence and unmet needs. *European Urology*.

[3] Smaldone M. C., Egleston B., Hollingsworth J. M. (2017). Understanding treatment disconnect and mortality trends in renal cell carcinoma using tumor registry data. *Medical Care*.

[4] Zhao Z., Liu H., Hou J. (2017). Tumor protein D52 (TPD52) inhibits growth and metastasis in renal cell carcinoma cells through the PI3K/Akt signaling pathway. *Oncology Research : Featuring Preclinical and Clinical Cancer Therapeutics*.

[5] Zhao H., Nolley R., Chan A. M. W., Rankin E. B., Peehl D. M. (2017). Cabozantinib inhibits tumor growth and metastasis of a patient-derived xenograft model of papillary renal cell carcinoma with MET mutation. *Cancer Biology & Therapy*.

[6] Cheah F. K., Leong K. H., Thomas N. F., Chin H. K., Ariffin A., Awang K. (2018). Resveratrol analogue, (E)-N-(2-(4-methoxystyryl) phenyl) furan-2-carboxamide induces G2/M cell cycle arrest through the activation of p53–p21CIP1/WAF1 in human colorectal HCT116 cells. *Apoptosis*.

[7] Huang X. Z., Kang L. P., Gao L., Zhang Y., Guo L. P., Huang L. Q. (2017). Quantative analysis of eupatilin and jaceosidin in folium of Artemisia argyi from different areas in China by RP-HPLC based on ancient medicine books. *Zhongguo Zhong Yao Za Zhi*.

[8] Lee J. H., Lee Y. J., Lee J. Y., Park Y. M. (2017). Topical application of eupatilin ameliorates atopic dermatitis-like skin lesions in NC/Nga mice. *Annals of Dermatology*.

[9] Lee H. M., Jang H. J., Kim S. S. (2016). Protective effect of eupatilin pretreatment against hepatic ischemia-reperfusion injury in mice. *Transplantation Proceedings*.

[10] Yu K., Li X. M., Xu X. L., Zhang R. Y., Cong H. L. (2015). Eupatilin protects against tumor necrosis factor-alpha-mediated inflammation inhuman umbilical vein endothelial cells. *International Journal of Clinical and Experimental Medicine*.

[11] Cho J.-H., Lee J.-G., Yang Y.-I. (2011). Eupatilin, a dietary flavonoid, induces G2/M cell cycle arrest in human endometrial cancer cells. *Food and Chemical Toxicology*.

[12] Zhong W.-F., Wang X.-H., Pan B., Li F., Kuang L., Su Z.-X. (2016). Eupatilin induces human renal cancer cell apoptosis via ROS-mediated MAPK and PI3K/AKT signaling pathways. *Oncology Letters*.

[13] Yang L., Lin Z., Wang Y. (2018). MiR-5100 increases the cisplatin resistance of the lung cancer stem cells by inhibiting the Rab6. *Molecular Carcinogenesis*.

[14] Wang L., He J., Xu H., Xu L., Li N. (2016). MiR-143 targets CTGF and exerts tumor-suppressing functions in epithelial ovarian cancer. *American Journal of Translational Research*.

[15] Huang H., Jiang Y., Wang Y. (2015). MiR-5100 promotes tumor growth in lung cancer by targeting Rab6. *Cancer Letters*.

[16] Patel H. D., Gorin M. A., Gupta N. (2016). Mortality trends and the impact of lymphadenectomy on survival for renal cell carcinoma patients with distant metastasis. *Canadian Urological Association Journal*.

[17] Cai M., Phan P.-T. T., Hong J. G. (2012). The neuroprotective effect of eupatilin against ischemia/reperfusion-induced delayed neuronal damage in mice. *European Journal of Pharmacology*.

[18] Li Y.-Y., Wu H., Dong Y.-G., Lin B., Xu G., Ma Y.-B. (2015). Application of eupatilin in the treatment of osteosarcoma. *Oncology Letters*.

[19] Cheong J.-H., Hong S. Y., Zheng Y., Noh S. H. (2011). Eupatilin inhibits gastric cancer cell growth by blocking STAT3-mediated VEGF expression. *Gastric Cancer*.

[20] Park B. B., Yoon J. S., Kim E. S. (2013). Inhibitory effects of eupatilin on tumor invasion of human gastric cancer MKN-1 cells. *Tumor Biology*.

[21] Rausch S., Beermann J., Scharpf M. (2016). Differential expression and clinical relevance of MUC1 in renal cell carcinoma metastasis. *World Journal of Urology*.

[22] Dong J., Cong L., Zhang T., Zhao Y. (2016). Pancreatic metastasis of renal cell carcinoma. *Hepatobiliary & Pancreatic Diseases International*.

[23] Liu R., Huang S., Lei Y. (2015). FGF8 promotes colorectal cancer growth and metastasis by activating YAP1. *Oncotarget *.

[24] Zhou R., Wang C., Wen C., Wang D. (2017). miR-21 promotes collagen production in keloid via Smad7. *Burns*.

[25] Chen D., Wang Z. (2017). Adrenaline inhibits osteogenesis via repressing miR-21 expression. *Cell Biology International*.

[26] Ju L., Zhou Z., Jiang B., Lou Y., Zhang Z. (2017). miR-21 is involved in skeletal deficiencies of zebrafish embryos exposed to polychlorinated biphenyls. *Environmental Science and Pollution Research*.

[27] Eseberri I., Lasa A., Miranda J., Gracia A., Portillo M. P. (2017). Potential miRNA involvement in the anti-adipogenic effect of resveratrol and its metabolites. *PLoS ONE*.

[28] Kuser-Abali G., Alptekin A., Lewis M., Garraway I. P., Cinar B. (2015). YAP1 and AR interactions contribute to the switch from androgen-dependent to castration-resistant growth in prostate cancer. *Nature Communications*.

[29] Ooki A., Del Carmen Rodriguez Pena M., Marchionni L. (2018). YAP1 and COX2 coordinately regulate urothelial cancer stem-like cells. *Cancer Research*.

[30] Wu D.-W., Lin P.-L., Wang L., Huang C.-C., Lee H. (2017). The YAP1/SIX2 axis is required for DDX3-mediated tumor aggressiveness and cetuximab resistance in KRAS-wild-type colorectal cancer. *Theranostics*.

[31] Celano M., Mignogna C., Rosignolo F. (2018). Expression of YAP1 in aggressive thyroid cancer. *Endocrine Journal*.

[32] Cai Y., Fu X., Deng Y. (2017). Histone demethylase JMJD1C regulates esophageal cancer proliferation Via YAP1 signaling. *American Journal of Cancer Research*.

[33] Huang C. Y., Han Z., Li X., Xie H. H., Zhu S. S. (2018). Inhibition of thyroid carcinoma cells with YAP1 protein interference and its mechanism. *European Review for Medical and Pharmacological Sciences*.

